# Tetra­ethyl­ammonium trichlorido(*N*,*N*′-di­methyl­formamide-κ*O*)zinc

**DOI:** 10.1107/S1600536813014323

**Published:** 2013-05-31

**Authors:** Guo-Ping Chao, Hua-Tian Shi, Qun Chen, Qian-Feng Zhang

**Affiliations:** a Department of Applied Chemistry, School of Petrochemical Engineering, Changzhou University, Jiangsu 213164, People’s Republic of China; b Institute of Molecular Engineering and Applied Chemistry, Anhui University of Technology, Ma’anshan, Anhui 243002, People’s Republic of China

## Abstract

The title complex salt, (C_8_H_20_N)[ZnCl_3_(C_3_H_7_NO)], contains one [Et_4_N]^+^ cation (Et is ethyl) and one [ZnCl_3_(DMF)]^−^ anion (DMF is di­methyl­formamide). In the anion, the zinc atom is tetra­hedrally coordinated by a DMF ligand *via* the O atom and by three terminal Cl atoms. The average Zn—Cl bond length and Cl—Zn—Cl angle are 2.243 (11) Å and 114 (3)°, respectively.

## Related literature

For background to zinc complexes: see: Folting *et al.* (1984 ▶); Gavens *et al.* (1982 ▶); For related structures, see: Bottomley *et al.* (1989 ▶); Hsu *et al.* (2008 ▶); Price *et al.* (1998 ▶); Shevchenko *et al.* (2008 ▶); For a description of the Cambridge Structural Database, see: Allen (2002 ▶). For standard bond lengths, see: Allen *et al.* (1987 ▶).
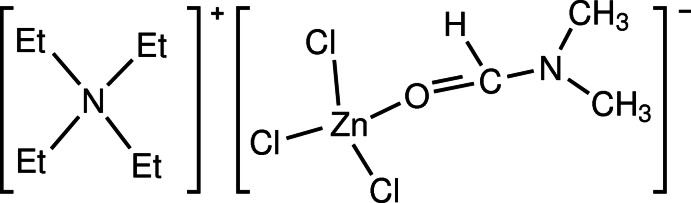


## Experimental

### 

#### Crystal data

(C_8_H_20_N)[ZnCl_3_(C_3_H_7_NO)]
*M* *_r_* = 375.07
Monoclinic, 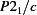

*a* = 12.3250 (18) Å
*b* = 8.8409 (13) Å
*c* = 16.721 (2) Å
β = 98.558 (2)°
*V* = 1801.7 (5) Å^3^
*Z* = 4
Mo *K*α radiation
μ = 1.80 mm^−1^
*T* = 296 K
0.29 × 0.20 × 0.16 mm

#### Data collection

Bruker APEXII CCD area-detector diffractometer
Absorption correction: multi-scan (*SADABS*; Sheldrick, 1997 ▶) *T*_min_ = 0.623, *T*_max_ = 0.762
10673 measured reflections
4047 independent reflections
3310 reflections with *I* > 2σ(*I*)
*R*_int_ = 0.024

#### Refinement

*R*[*F* ^2^ > 2σ(*F* ^2^)] = 0.031
*wR*(*F* ^2^) = 0.078
*S* = 1.04
4047 reflections
169 parameters
H-atom parameters constrained
Δρ_max_ = 0.46 e Å^−3^
Δρ_min_ = −0.53 e Å^−3^

### 

Data collection: *APEX2* (Bruker, 2005 ▶); cell refinement: *SAINT* (Bruker, 2005 ▶); data reduction: *SAINT*; program(s) used to solve structure: *SHELXS97* (Sheldrick, 2008 ▶); program(s) used to refine structure: *SHELXL97* (Sheldrick, 2008 ▶); molecular graphics: *SHELXTL* (Sheldrick, 2008 ▶); software used to prepare material for publication: *SHELXTL*.

## Supplementary Material

Crystal structure: contains datablock(s) I, global. DOI: 10.1107/S1600536813014323/ds2232sup1.cif

Structure factors: contains datablock(s) I. DOI: 10.1107/S1600536813014323/ds2232Isup2.hkl

Additional supplementary materials:  crystallographic information; 3D view; checkCIF report

## Figures and Tables

**Fig. 1.**
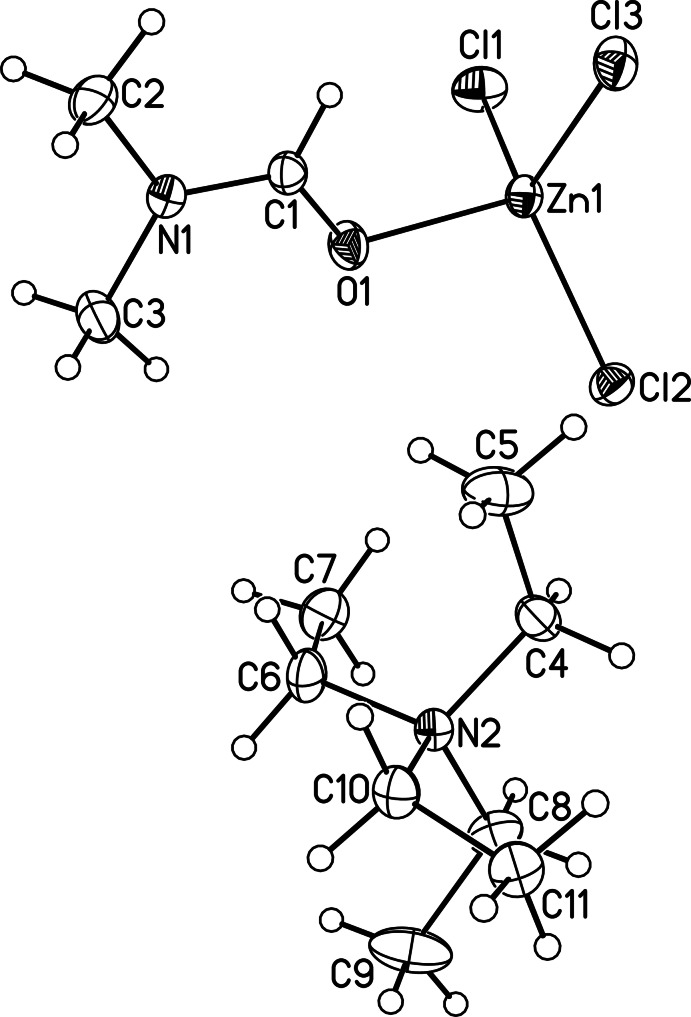
The structure of the title compound, showing the atom-numbering scheme and displacement ellipsoids at the 50% probability level.

**Fig. 2.**
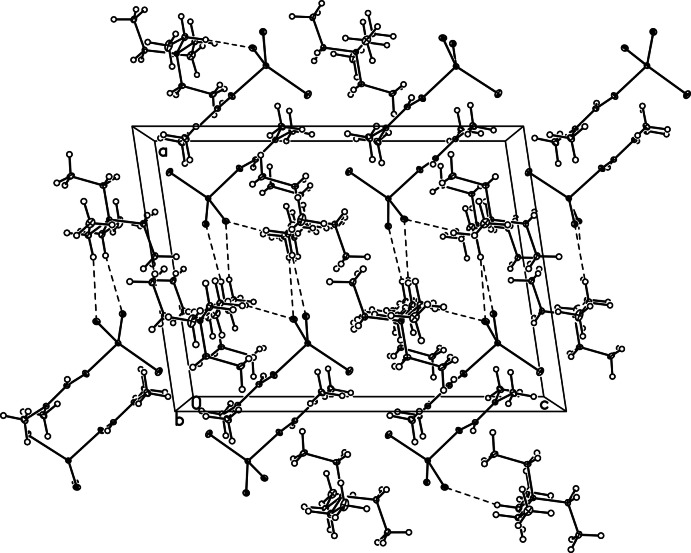
Packing diagram of the title compound in a unit cell.

## supplementary crystallographic information

### Comment

Inclusion of electropositive metal ions such as A1^3+^, Mg^2+^, and Zn^2+^ in recipes for Ziegler-Natta olefin polymerization catalysts can substantially alter catalyst performance (Gavens *et al.*, 1982). For example, the reactivity of ZnC1_2_ toward early-transition-metal halides may yield a series of new materials for the function of co-catalysts (Folting *et al.*, 1984). Actually, the direct reaction of ZnCl_2_ with other transition metal compounds has obvious limitation due to its solubility, it is necessary to develop new organometallic reagent of zinc metal. The reaction of anhydrous ZnCl_2_ with equal equivalent of [Et_4_N]Cl in DMF yielded soluble [Et_4_N][ZnCl_3_(DMF)] which was structurally characterized in this paper.

The title compound crystallizes in the monoclinic crystal system, containing two independent ions: [Et_4_N]^+^ cation and [ZnCl_3_(DMF)]^-^ anion. The molecular structure of the title compound is depicted in Fig. 1. In [ZnCl_3_(DMF)]^-^ anion, the zinc atom adopts a distorted tetrahedral geometry with a {three chloride and one oxygen} donor set involving the carbonyl oxygen O(1) of the *N*,*N*'-dimethylformide. Bond lengths to the metal atom are Zn(1)—Cl(1) 2.2397 (6), Zn(1)—Cl(2) 2.2332 (6), Zn(1)—Cl(3) 2.2552 (6) [av. Zn(1)—Cl = 2.2427 (6) Å] and Zn(1)—O(1) 2.0482 (14) Å, which agree well with those in [ZnCl_3_(LH)] (L = 1-(2-hydroxyethyl)-(2-aminoethyl-N^2^)-5-methylisocytosine) (Price *et al.*, 1998) and [Co(L1)Cl_2_][ZnCl_3_(DMF)] (L1 = 4,6,6-trimethyl-1,9-diamino-3,7-diazanon-3-ene) (Shevchenko *et al.*, 2008). The tetrahedral coordination geometry around the zinc atom is considerably distorted, which is indicated by the Cl—Zn—Cl and O—Zn—Cl angles being in the ranges 104.46 (2)—116.71 (2) and 101.98 (5)—104.46 (5)°, respectively. The Zn—O bond length in the title compound (2.0482 (14) Å) is compared with those in (C_13_H_28_N_2_)_2_[Zn_2_(C_8_H_4_O_4_)Cl_6_].4H_2_O (1.956 (3) Å) (Hsu *et al.*, 2008), [ZnCl(C_4_H_8_O)(µ-Cl)]_n_ (1.981 (3) Å) (Bottomley *et al.*, 1989), and [Co(L1)Cl_2_][ZnCl_3_(DMF)] (L1 = 4,6,6-trimethyl-1,9-diamino-3,7-diazanon-3-ene) (2.01 (1) Å) (Shevchenko *et al.*, 2008). The [Et_4_N]^+^ cation in the title compound has its expected structure as well as normal distances and angles, which will not be discussed further (Allen *et al.*, 1987).

### Experimental

Equal equivalent of anhydrous ZnCl_2_ and [Et_4_N]Cl was mixed in DMF, the mixture was stirred for 2 h at room temperature, and then Et_2_O was added slowly to clearly colourless solution and the precipitate that formed was filtered off with suction, washed with Et_2_O three times and dried in vacuo, yielding a white solid in ca. 94 %. Colourless crystals were obtained by slow diffusion of Et_2_O into a DMF solution of the white powder over several days. Anal. Calcd. for C_11_H_27_N_2_OCl_3_Zn: C, 35.22; H, 7.26; N, 7.47%. Found: C, 35.15; H, 7.22; N, 7.43%.

### Refinement

The structure was solved by direct methods and refined by full-matrix least-squares procedure based on F^2^. All C Hydrogen atoms were placed in geometrically idealized positions and refined isotropically with a riding model for both C-sp^2^ [C—H = 0.93 Å and with *U*_iso_(H) = 1.2*U*_eq_(C)] and C-sp^3^ [C—H = 0.96—0.97 Å and with *U*_iso_(H) = 1.5*U*_eq_(C)].

### Crystal data

**Table:**

(C_8_H_20_N)[ZnCl_3_(C_3_H_7_NO)]	*F*(000) = 784
*M**_r_* = 375.07	*D*_x_ = 1.383 Mg m^−^^3^
Monoclinic, *P*2_1_/*c*	Mo *K*α radiation, λ = 0.71073 Å
Hall symbol: -P 2ybc	Cell parameters from 4191 reflections
*a* = 12.3250 (18) Å	θ = 2.5–27.1°
*b* = 8.8409 (13) Å	µ = 1.80 mm^−^^1^
*c* = 16.721 (2) Å	*T* = 296 K
β = 98.558 (2)°	Block, colourless
*V* = 1801.7 (5) Å^3^	0.29 × 0.20 × 0.16 mm
*Z* = 4	

### Data collection

**Table:**

Bruker APEXII CCD area-detector diffractometer	4047 independent reflections
Radiation source: fine-focus sealed tube	3310 reflections with *I* > 2σ(*I*)
Graphite monochromator	*R*_int_ = 0.024
phi and ω scans	θ_max_ = 27.5°, θ_min_ = 2.8°
Absorption correction: multi-scan (*SADABS*; Sheldrick, 1997)	*h* = −15→15
*T*_min_ = 0.623, *T*_max_ = 0.762	*k* = −6→11
10673 measured reflections	*l* = −21→18

### Refinement

**Table:**

Refinement on *F*^2^	Primary atom site location: structure-invariant direct methods
Least-squares matrix: full	Secondary atom site location: difference Fourier map
*R*[*F*^2^ > 2σ(*F*^2^)] = 0.031	Hydrogen site location: inferred from neighbouring sites
*wR*(*F*^2^) = 0.078	H-atom parameters constrained
*S* = 1.04	*w* = 1/[σ^2^(*F*_o_^2^) + (0.0411*P*)^2^ + 0.2235*P*] where *P* = (*F*_o_^2^ + 2*F*_c_^2^)/3
4047 reflections	(Δ/σ)_max_ = 0.001
169 parameters	Δρ_max_ = 0.46 e Å^−^^3^
0 restraints	Δρ_min_ = −0.53 e Å^−^^3^

### Special details

**Table:**

Geometry. All esds (except the esd in the dihedral angle between two l.s. planes) are estimated using the full covariance matrix. The cell esds are taken into account individually in the estimation of esds in distances, angles and torsion angles; correlations between esds in cell parameters are only used when they are defined by crystal symmetry. An approximate (isotropic) treatment of cell esds is used for estimating esds involving l.s. planes.
Refinement. Refinement of F^2^ against ALL reflections. The weighted R-factor wR and goodness of fit S are based on F^2^, conventional R-factors R are based on F, with F set to zero for negative F^2^. The threshold expression of F^2^ > 2sigma(F^2^) is used only for calculating R-factors(gt) etc. and is not relevant to the choice of reflections for refinement. R-factors based on F^2^ are statistically about twice as large as those based on F, and R- factors based on ALL data will be even larger.

### Fractional atomic coordinates and isotropic or equivalent isotropic displacement parameters (Å^2^)

**Table:**

	*x*	*y*	*z*	*U*_iso_*/*U*_eq_	
Zn1	0.768184 (17)	0.26604 (3)	0.138235 (13)	0.04102 (9)	
Cl1	0.86974 (6)	0.22428 (7)	0.03995 (4)	0.06676 (17)	
Cl2	0.66678 (4)	0.47639 (6)	0.12929 (3)	0.05385 (14)	
Cl3	0.68562 (4)	0.05121 (6)	0.17003 (3)	0.05772 (15)	
O1	0.88052 (12)	0.30791 (17)	0.23893 (8)	0.0539 (4)	
N1	0.99317 (13)	0.22084 (17)	0.34825 (9)	0.0408 (3)	
N2	0.67697 (12)	0.71220 (18)	0.39112 (9)	0.0373 (3)	
C1	0.91867 (15)	0.2034 (2)	0.28445 (11)	0.0426 (4)	
H1	0.8924	0.1062	0.2723	0.051*	
C2	1.03514 (19)	0.0923 (3)	0.39750 (14)	0.0609 (6)	
H2A	0.9998	0.0016	0.3755	0.091*	
H2B	1.0205	0.1065	0.4518	0.091*	
H2C	1.1129	0.0840	0.3978	0.091*	
C3	1.03536 (19)	0.3696 (3)	0.37379 (14)	0.0603 (6)	
H3A	0.9978	0.4453	0.3389	0.091*	
H3B	1.1125	0.3738	0.3708	0.091*	
H3C	1.0236	0.3879	0.4284	0.091*	
C4	0.61949 (17)	0.5862 (2)	0.33990 (13)	0.0513 (5)	
H4A	0.5418	0.5912	0.3436	0.062*	
H4B	0.6277	0.6048	0.2839	0.062*	
C5	0.6591 (2)	0.4277 (3)	0.36167 (19)	0.0823 (8)	
H5A	0.7345	0.4182	0.3540	0.123*	
H5B	0.6153	0.3563	0.3276	0.123*	
H5C	0.6527	0.4077	0.4172	0.123*	
C6	0.79920 (17)	0.7070 (3)	0.38967 (14)	0.0565 (5)	
H6A	0.8276	0.6129	0.4143	0.068*	
H6B	0.8333	0.7891	0.4228	0.068*	
C7	0.8331 (2)	0.7187 (3)	0.30666 (16)	0.0644 (6)	
H7A	0.8058	0.6327	0.2748	0.097*	
H7B	0.9117	0.7213	0.3118	0.097*	
H7C	0.8034	0.8096	0.2807	0.097*	
C8	0.62689 (19)	0.8583 (2)	0.35567 (13)	0.0553 (5)	
H8A	0.6373	0.8635	0.2994	0.066*	
H8B	0.5485	0.8551	0.3570	0.066*	
C9	0.6723 (3)	1.0009 (3)	0.39742 (17)	0.0973 (11)	
H9A	0.6589	1.0001	0.4525	0.146*	
H9B	0.6370	1.0872	0.3700	0.146*	
H9C	0.7498	1.0062	0.3963	0.146*	
C10	0.66162 (17)	0.6958 (3)	0.47938 (11)	0.0500 (5)	
H10A	0.6899	0.5979	0.4986	0.060*	
H10B	0.7057	0.7723	0.5106	0.060*	
C11	0.54520 (19)	0.7095 (3)	0.49624 (13)	0.0574 (6)	
H11A	0.5174	0.8083	0.4807	0.086*	
H11B	0.5436	0.6946	0.5529	0.086*	
H11C	0.5004	0.6343	0.4658	0.086*	

### Atomic displacement parameters (Å^2^)

**Table:**

	*U*^11^	*U*^22^	*U*^33^	*U*^12^	*U*^13^	*U*^23^
Zn1	0.03799 (13)	0.04580 (15)	0.03841 (13)	−0.00102 (9)	0.00288 (9)	0.00403 (9)
Cl1	0.0766 (4)	0.0652 (4)	0.0659 (4)	0.0039 (3)	0.0349 (3)	−0.0006 (3)
Cl2	0.0537 (3)	0.0524 (3)	0.0547 (3)	0.0098 (2)	0.0054 (2)	0.0019 (2)
Cl3	0.0477 (3)	0.0563 (3)	0.0674 (3)	−0.0095 (2)	0.0027 (2)	0.0181 (3)
O1	0.0539 (8)	0.0539 (9)	0.0486 (8)	−0.0036 (7)	−0.0099 (6)	0.0072 (7)
N1	0.0372 (8)	0.0447 (9)	0.0398 (8)	0.0018 (7)	0.0037 (6)	0.0020 (7)
N2	0.0352 (8)	0.0417 (8)	0.0350 (7)	−0.0015 (6)	0.0050 (6)	0.0012 (6)
C1	0.0365 (10)	0.0467 (11)	0.0443 (10)	−0.0015 (8)	0.0047 (8)	−0.0015 (8)
C2	0.0649 (14)	0.0563 (13)	0.0574 (13)	0.0154 (11)	−0.0050 (10)	0.0062 (11)
C3	0.0624 (13)	0.0561 (13)	0.0572 (13)	−0.0133 (11)	−0.0087 (10)	0.0020 (10)
C4	0.0497 (11)	0.0536 (12)	0.0513 (11)	−0.0106 (10)	0.0103 (9)	−0.0124 (10)
C5	0.105 (2)	0.0462 (14)	0.103 (2)	−0.0076 (14)	0.0391 (17)	−0.0047 (14)
C6	0.0352 (10)	0.0756 (15)	0.0582 (13)	−0.0051 (10)	0.0055 (9)	−0.0006 (11)
C7	0.0483 (13)	0.0777 (17)	0.0728 (16)	−0.0048 (11)	0.0274 (11)	−0.0004 (12)
C8	0.0700 (14)	0.0486 (12)	0.0504 (11)	0.0135 (11)	0.0190 (10)	0.0112 (10)
C9	0.170 (3)	0.0429 (14)	0.089 (2)	−0.0056 (17)	0.055 (2)	−0.0052 (13)
C10	0.0515 (12)	0.0628 (13)	0.0356 (10)	−0.0011 (10)	0.0056 (8)	0.0050 (9)
C11	0.0590 (13)	0.0689 (15)	0.0474 (12)	−0.0043 (11)	0.0184 (10)	0.0022 (10)

### Geometric parameters (Å, º)

**Table:**

Zn1—O1	2.0482 (14)	C5—H5A	0.9600
Zn1—Cl2	2.2332 (6)	C5—H5B	0.9600
Zn1—Cl1	2.2397 (6)	C5—H5C	0.9600
Zn1—Cl3	2.2552 (6)	C6—C7	1.512 (3)
O1—C1	1.244 (2)	C6—H6A	0.9700
N1—C1	1.309 (2)	C6—H6B	0.9700
N1—C2	1.452 (3)	C7—H7A	0.9600
N1—C3	1.455 (3)	C7—H7B	0.9600
N2—C6	1.511 (2)	C7—H7C	0.9600
N2—C8	1.514 (2)	C8—C9	1.508 (3)
N2—C4	1.515 (2)	C8—H8A	0.9700
N2—C10	1.523 (2)	C8—H8B	0.9700
C1—H1	0.9300	C9—H9A	0.9600
C2—H2A	0.9600	C9—H9B	0.9600
C2—H2B	0.9600	C9—H9C	0.9600
C2—H2C	0.9600	C10—C11	1.508 (3)
C3—H3A	0.9600	C10—H10A	0.9700
C3—H3B	0.9600	C10—H10B	0.9700
C3—H3C	0.9600	C11—H11A	0.9600
C4—C5	1.511 (3)	C11—H11B	0.9600
C4—H4A	0.9700	C11—H11C	0.9600
C4—H4B	0.9700		
			
O1—Zn1—Cl2	101.98 (5)	C4—C5—H5C	109.5
O1—Zn1—Cl1	104.46 (5)	H5A—C5—H5C	109.5
Cl2—Zn1—Cl1	117.16 (2)	H5B—C5—H5C	109.5
O1—Zn1—Cl3	103.41 (4)	N2—C6—C7	115.17 (19)
Cl2—Zn1—Cl3	116.71 (2)	N2—C6—H6A	108.5
Cl1—Zn1—Cl3	110.80 (2)	C7—C6—H6A	108.5
C1—O1—Zn1	121.10 (14)	N2—C6—H6B	108.5
C1—N1—C2	121.17 (17)	C7—C6—H6B	108.5
C1—N1—C3	121.48 (17)	H6A—C6—H6B	107.5
C2—N1—C3	117.33 (17)	C6—C7—H7A	109.5
C6—N2—C8	111.66 (15)	C6—C7—H7B	109.5
C6—N2—C4	110.61 (15)	H7A—C7—H7B	109.5
C8—N2—C4	106.07 (16)	C6—C7—H7C	109.5
C6—N2—C10	106.30 (14)	H7A—C7—H7C	109.5
C8—N2—C10	111.01 (15)	H7B—C7—H7C	109.5
C4—N2—C10	111.27 (15)	C9—C8—N2	115.5 (2)
O1—C1—N1	124.53 (19)	C9—C8—H8A	108.4
O1—C1—H1	117.7	N2—C8—H8A	108.4
N1—C1—H1	117.7	C9—C8—H8B	108.4
N1—C2—H2A	109.5	N2—C8—H8B	108.4
N1—C2—H2B	109.5	H8A—C8—H8B	107.5
H2A—C2—H2B	109.5	C8—C9—H9A	109.5
N1—C2—H2C	109.5	C8—C9—H9B	109.5
H2A—C2—H2C	109.5	H9A—C9—H9B	109.5
H2B—C2—H2C	109.5	C8—C9—H9C	109.5
N1—C3—H3A	109.5	H9A—C9—H9C	109.5
N1—C3—H3B	109.5	H9B—C9—H9C	109.5
H3A—C3—H3B	109.5	C11—C10—N2	115.63 (17)
N1—C3—H3C	109.5	C11—C10—H10A	108.4
H3A—C3—H3C	109.5	N2—C10—H10A	108.4
H3B—C3—H3C	109.5	C11—C10—H10B	108.4
C5—C4—N2	116.02 (19)	N2—C10—H10B	108.4
C5—C4—H4A	108.3	H10A—C10—H10B	107.4
N2—C4—H4A	108.3	C10—C11—H11A	109.5
C5—C4—H4B	108.3	C10—C11—H11B	109.5
N2—C4—H4B	108.3	H11A—C11—H11B	109.5
H4A—C4—H4B	107.4	C10—C11—H11C	109.5
C4—C5—H5A	109.5	H11A—C11—H11C	109.5
C4—C5—H5B	109.5	H11B—C11—H11C	109.5
H5A—C5—H5B	109.5		
